# Report on the Progress of Anatomy and Physiology

**Published:** 1846-01

**Authors:** W. H. Ranking


### RECORD OF MEDICAL SCIENCE.

### *Report on the Progress of Anatomy and Physiology.*

By W. H. Ranking, M. D.

### § 1.—*The Blood.*

1. Dr. G. O. Rees has recently offered some new views concerning the physical and physiological attributes of the blood.

1st. He has proved beyond doubt that the red corpuscles are vesicular, as is now generally supposed. Their vesicular character is clearly shown in the readiness with which they become collapsed or distended by increasing or diminishing the specific gravity of the medium in which they float. In order to collapse the corpuscles, a solution of sp. gr. 1060 is sufficient, but a solution of 1070 or more is required to produce a decided effect. Solutions cease to distend the corpuscles when of sp. gr. 1050 to 55, and to distend them when well a solution of 1015 or 10 is desirable. The sp. gr. of blood is about 1057 to 60; and since the corpuscles remain unaltered by solutions of from 1050 to 60, it may be concluded that the average sp. gr. of liquor sanguinis lies somewhere between these two points; and this proves that the fibrine of the blood is dissolved, and not merely suspended in the liquor sanguinis.

2d. He has also rendered decisive the question as to where the red colouring matter of the corpuscles is seated, proving it to be contained in the fluid within the vesicle, and that the envelopes themselves are white or colourless membranes. This is shown by increasing the sp. gr. of the liquid in which the corpuscles float, the result of which is the escape by *exosmosis* of the red coloured fluid from within the corpuscles; and again by applying water to the corpuscles, and so inducing *endosmosis*, the vesicles become distended and burst, their colouring matter mixes with the water, and their envelopes subside to the bottom of the vessel, forming a white layer.

3d. By examining the white layer deposited in the manner just mentioned (in which, together with *granules* and shreds of membrane, are found numerous white bodies resembling blood corpuscles, though smaller; and from analogy, he entertains no doubt that the red corpuscles contain nuclei, which however are so highly refractive of light, that they are not distinguishable in the corpuscles themselves. He describes the nucleus as being about $\frac{1}{4500}$ of an inch in diameter, or $\frac{3}{2}$ the size of the red corpuscles, in the centre of which it is situated; it is flattened and circular like the corpuscle, though it differs from it in not being rounded at its edge; it is adherent to the envelope only at its centre, leaving a canal all round its free edge, which canal contains the red colouring matter.

4th. The whole of the iron of the blood is contained in the red colouring matter; the envelopes and nuclei do not present a trace of it. The corpuscles obtain their iron from the chyle, the serum of which holds a large quantity of it in solution; the sp. gr. of the chyle is considerably lower than that of liquor sanguinis; consequently, when the former becomes mixed with the blood, it dilutes the liquor sanguinis, and so induces an endosmodic current rich in iron to enter the corpuscles. How the colour of the contents of the corpuscles is produced remains a mystery; it is within the red corpuscles alone that it is effected, there is no red colouring matter in lymph or chyle; any admixture of red corpuscles found in these latter is an accidental circumstance attending the mode of examination.

5th. Dr. Rees seems to admit the probability of the exudation and fibrinous corpuscles observed so abundantly in effused coagulable lymph, having their origin in the pale corpuscles of the blood, for he sees no difficulty in these pale corpuscles passing through pores in the blood-vessels which would not admit the red corpuscles; the latter he compares to bladders filled with fluid, and which are not capable of yielding until their membrane be ruptured, but the former being soft solids may be compressed like a sponge, and pass through an opening, the orifice of which is smaller than the corpuscles themselves.

6th. He justly condemns the coarse and necessarily inaccurate method hitherto pursued in the quantitative analysis of blood. Amongst other fallacies attending the present method, the corpuscles are estimated as hematosine alone, no allowance being made for the envelopes or nuclei. Again, a certain quantity ot fibrine will be estimated as hematosine, because, as the fibrine of the liquor sanguinis coagulates, the sp. gr. of the medium in which the corpuscles float, becomes lowered, and the corpuscles will thus draw into their interior more liquor sanguinis, and so absorb a certain quantity of fibrine with which they will subside, When the fibrine coagulates slowly, the quantity thus disposed of will be large, for each of the immense number of corpuscles will absorb a little. Again, *all* the water of the corpuscles should not be estimated as belonging to serum, because, although the corpuscles must have endosmosed some serum during coagulation of the liquor sanguinis ; yet, in their natural condition they contain a fluid which is quite distinct from serum. To obtain pure hematosine by a method free from these fallacies, Dr. Rees recommends the corpuscles to be cleaned by repeated washings in a solution of salt and water, or sugar and water, the sp. gr. of which is equal to that of the liquor sanguinis, so that the corpuscles may be unaffected by endosmosis. When thus cleansed, they are to be moved into a vessel of water, the result of which is, that the individual corpuscles swell and burst, their colouring matter is discharged into the water, whilst the nuclei and envelopes subside as a precipitate ; both ingredients are thus in a fit condition for estimation and further examination.

7th. In regard to *genesis*, or the original formation of blood-corpuscles, he considers that they multiply by division, for on examining a portion of blood maintained at about its natural temperature, he observed the corpuscles to assume an hour-glass form, which increasing, eventually divided each corpuscle into two unequal sized circular bodies, which, when treated with a strong saline solution, underwent the same exosmotic changes as observed in common blood-corpuscles. When worn out, the blood-corpuscles become disintegrated and their debris exist floating in the liquor sanguinis. By diluting the serum of coagulated blood with water, a precipitate forms, consisting of the envelopes and nuclei of old corpuscles disintegrated ; this disposal of the worn out corpuscles will account for the traces of iron occasionally found in the serum.

8th. Dr. Rees considers the explanation offered by Mülder regarding the mode in which the change of colour from dark to bright red is effected in the blood during its passage through the lungs, to be entirely hypothetical and erroneous. Mülder accounts for the change of colour from venous to arterial, and from arterial to venous blood in this way. He supposes that " in traversing the capillary system of the lungs, the proteine of the blood combines with oxygen, generating a compound analogous to 'bufly-coat,' which forms a contractile covering to the blood-corpuscles, causing them to become more opaque, and giving them the figure of doubly concave lenses; that in the general capillary circulation, this layer of oxyproteine surrounding the corpuscles is decomposed, the oxygen being used for dissolving old tissue, and the proteine deposited to supply its place. The corpuscles lose their reflecting concave figure, and become more transparent by this change. That the difference of colour between arterial and venous blood depends solely upon a physical difference in the surfaces of the corpuscles, being semi-opaque concave mirrors in the former, and more transparent convex bodies in the latter; and that during respiration the colouring matter itself of the blood undergoes no change, and indeed plays no part, either as a whole, or in regard to the iron which exists merely as a simple element in it." To these several views Dr. Rees is entirely opposed; he rejects the idea of a layer of plastic oxyproteine being deposited on the blood-corpuscles during respiration, and instead of considering the hematosine as undergoing no change, and maintaining the same condition in arterial and venous blood, he looks upon it as being the cause of the change in the colour of the blood in virtue of some chemical alteration which take place in it. In this view he agrees with Liebig, though he does not adopt the explanation offered by this chemist as to the nature of this alteration. According to Liebig, the change in colour observed to take place during the passage of venous blood through the lungs is due to the formation of a carbonate of the peroxide of iron in the red corpuscles, which, during the passage of arterial blood through the system, is resolved into carbonate of the protoxide, by the abstraction of oxygen for the gènerel purposes of the system, and thus the dark colour is again restored to the blood. This hypothesis is mainly disproved by the impossibility of obtaining any trace of an oxide of iron from the red corpuscles by treating them with a weak acid, by there being no difference in the quantity of oxygen in arterial and venous blood, and by all the iron admitting of extraction from the hematosine, without the other chemical constituents of the hematosine being interfered with, and especially without any diminution in the quantity of its oxygen being effected. What may be the nature of this change, therefore in the hematosine [if any does really exist] on which the alteration in the colour of the blood depends, remains still a mystery.

Having accounted for the change in colour in the blood whilst in the body, by the alternate deposition and removal of a layer of plastic material by which an alteration in the form of the corpuscle is produced, and the change in colour follows as an optical effect, Mülder proceeded to explain in a similar manner the changes in colour undergone by the blood under various circumstances out of the body, such as result from exposure to the action of saline solutions of water and other reagents; in all these cases he considers that an alteration in the figure of the corpuscle is the cause of the change in colour observed, and that anything which produces this alteration in figure is capable of modifying the tint of the blood. Thus he considers that solutions of salt render a coagulum of blood of a bright red colour by exosmosing the corpuscles, and thus making them assume a biconcave form, and that when the corpuscles are endosmosed, and assume a convex form [as by washing the reddened coagulum in water] the colour of the blood is darkened in consequence; but Dr. Rees considers this statement to be incorrect, for saline matters will render a coagulum of a bright red colour. whether an endosmotic or an exosmotic action be induced; besides, blood-corpuscles may be changed in form, without any variation in the colour of the blood being produced in consequence. Moreover, strong saline solutions cause the corpuscles to become flaccid and empty, and not to assume that biconcave form which Mülder considers so well calculated for the reflexion of light. Mülder and others conceive that the iron in the hematosine has nothing to do with the colour of the blood, inasmuch as this metallic ingredient may be removed without the colour being destroyed; Dr. Rees objects to this being received as a conclusive argument against the iron being essential to the formation of the red colour.

2. *Formation of the buffy coat.* Mr. Gulliver has given the results of some experiments on the coagulation of the blood, which seem to add confirmation to the view entertained by Mr. Wharton Jones and others, that the formation of the buffy coat is due in great measure to an increased aggregation between the red corpuscles, by which these latter tend to arrange themselves in rolls, and consequently to subside to the bottom of the vessel more readily than they would do as individual corpuscles. With this tendency to rapid subsidence there is also usually combined a slowness in the coagulation of the liquor sanguinis, so that the corpuscles have time to leave the upper layers before coagulation commences. Mr. Hewson and Dr. Davy, however, maintain, that the rapid sinking of the corpuscles is due to an attenuation of the liqnor sanguinis, but against this Mr. Gulliver argues, that if we admit the sinking of the red corpuscles to afford an accurate test of the consistency of the liquor sanguinis, we must also admit what seems improbable, that the liquor sanguinis becomes thinner some minutes after the blood has been withdrawn, for at that time the falling of the red corpuscles is most rapid. Following are some of Mr. Gulliver's conclusions:—

1st. There is a remarkable acceleration after a few minutes, in the rate of which the blood-corpuscles sink into the liquor sanguinis. 2d. This acceleration may be increased by increasing the aggregation of the corpuscles; and prevented or reversed by preventing or destroying the aggregation of the corpuscles. 3d. The sinking of the corpuscles is slower in blood thickened by weak saline solutions, than when mucilage is added with the salt. 4th. The sinking of the corpuscles may be slower in serum artificially made thicker and heavier. 5th. In the cruor of horse's blood, the corpuscles are more aggregate, and have a greater appearance of agglutination, than in very buffy human blood. 6th. There may be a buffy coat, or only a comparatively thin one, in the blood of the horse, when the blood has been made thinner, and its coagulation retarded. 7th. The corpuscles of the horse sink much more quickly in his serum than the corpuscles of man do in his. 8th. Increasing the proportion of corpuscles in the blood hastens coagulation, and prevents or diminishes the formation of the buffy coat, more than increasing the serum only.

3. *Corpuscles.* Mr. Hobson has examined microscopically the blood-corpuscles of the ornithorhyncus, kangaroo, and phalanger. Those of the ornithorhyncus have the form of circular discs, like those of the most part of other mammalia, and their diameter differs but little from the diameter of the corpuscles of human blood, averaging about $\frac{1}{3000}$. of an inch. In the kangaroo, the corpuscles are somewhat smaller, and in the phalanger they are about $\frac{1}{3500}$ of an inch. The corpuscles of the eschidnè, which Mr. Hobson has also examined, resemble very closely those of the ornithorhyncus.

4. *Inflammation in cold-blooded animals.* Mr. Hereboullet gives the particulars of an examinatiou made on an alligator which had died of *peritonitis* occurring as a consequence of perforation of the intestine. Within the peritoneum were found all the characters of true inflammation; intense redness, effusions of lymph, false membranes, agglutination of the intestines and purulent secretion, proving beyond doubt the possibility of inflammation taking place in cold-blooded animals.

§ II.—*Digestion.*

5. *Vomiting.* Some observations on the subject of vomiting have been offered by Mr. Paget chiefly in reference to the agency of the diaphragm. The deep inspiration preceding the act of vomiting is terminated by the closure of the glottis; after this the diaphragm cannot move at all without expanding or compressing the air in the lungs, therefore it presents an unyielding surface against which the stomach may be pressed by the contracting abdominal muscles. Most probably, as observed by Mr. Anderson, the diaphragm remains during the act of vomiting in a state of rigid contraction, though were it even relaxed it would still present a resisting surface on account of the closure of the glottis. Another condition essential to vomiting is the relaxation of the oblique fibres of the stomach, which probably takes place exactly in the coincidence with the contraction of the abdominal muscles, just as the relaxation of the muscles closing the glottis occurs coincidently with the contractions of the expiratory muscles in the acts of coughing, sneezing, &c.

6, *Intestinal villi. Lacteal absorption*:—It has long been a matter of difficulty to explain how chyle, or the nutritive portion of chyme, found its way into the lacteals. The fancied existence of mouths or openings at the extremities of the lacteal tubes, as described by Mr. Cruikshank and Dr. William Hunter (and generally admitted during the period in which they wrote,) as explanatory of the mode in which the lacteals and lymphatics in general performed their functions, seemed to remove this difficulty; but the researches of Krause, Valentin, and others, having disproved the existence of orifices in the lacteals, the mystery of absorption remained as great as before.

Mr. Goodsir, a few years ago, was one of the first to suggest that the process of lacteal absorption is effected through the medium of cells; and by recent observations, he has proved almost beyond question that cells are the real agents by which the selection and absorption of the nutritive portions of the chyme take place. When viewed in this light, the matter is greatly simplified, especially since it is almost certain that nearly all the changes which are constantly occurring in the whole organic kingdom, on the grandest as well as the simplest scale, are effected through the agency of those minute, yet all-important bodies—nucleated cells. These cells seem endowed with a peculiar independent vitality, by means of which, at the time that they grow themselves, they are absorbing into their interior, from the surrounding medium, materials which they can convert either into the elements of tissue, as of muscle, nerve, bone, &c., if nutrition be the function assigned to them; or into some peculiar fluid of secretion, as of milk, bile, saliva, &c., if secretion be their especial object. Thus, growing themselves, they produce the growth of others, and by feeding themselves, they draw off waste materials from the body, and render them subservient to further useful purposes in the economy, or eject them from the system as refuse, by the natural outlets. The following comprises the substance of Mr. Goodsir's paper on the intestinal villi. 1. Each time chyme passes along the intestines, the villi receive an increased supply of blood; they become turgid and erect, and the epithelium covering them is cast off, so that the chyme can come into actual contact with their exposed surface. 2. At the same time, the epithelia lining the follicles of Lieberkühn are also thrown off, and mix with the chyme contained in the intestines; they probably contain a secreted fluid, subservient to the process of chylification. 3. Each villus, besides its one or two looped lacteals, and the minute network of blood-vessels lining its walls, contains, in its quiescent state, scattered amongst the terminal loops of the lacteals, numerous granular particles, which are the germs or nuclei of absorbing vesicles; during the process of chime absorption, these minute germs become gradually developed into vesicles, probably by deriving nutriment from the neighbouring blood-vessels. Whilst they increase in size, these vesicles, by virtue of their peculiar absorbing power, draw into their interior certain materials from the chyme surrounding the villi, which they probably elaborate; having attained their full size, they burst, and discharge their contents etther directly into the lacteals, or, more probably, into the texture of the villus, whence they are taken up by the lacteals. The function of the lacteals thus seems to consist in removing the debris and contents of the dissolved chyle cells. 4. Each villus is permanently covered (as also are the follicles of Lieberkühn lined) by a fine, smooth, germinal membrane, containing in its substance germinal centres of an oval form, situated at pretty regular distances, the office of which is the production of fresh epithelium cells to cover again and protect the surface of the villus, after the absorption of chyme is ended. 5. This mode of absorption by the chyle cells renders the analogy very striking between intestinal villi and the spongioles of plants; the latter of which most probably absorb nutriment for the plant through the medium of their cells. The soil in this latter case holds a relation towards the spongioles somewhat similar to that of chyme towards the villi. 6. It is probable that in the villi, as also in the spongioles of plants, the absorbed alimentary matters undergo the first steps of the organizing or vitalizing process.

The only difficulty which presents itself in the above theory of Mr. Goodsir, regarding the mode of absorption by the cells of the intestinal villi, is to understand how these cells can absorb materials for their own growth from the neighbouring blood-vessels, at the same time that they are also absorbing materials for the formation of chyle from the matters contained in the intestines; but this difficulty vanishes when we remember the wonderful endowments of cells in general, the power of selection and disposal of materials which they possess, together with other properties, showing that they are gifted with a peculiar independent vitality, to enable them to discharge certain important functions, the kinds of which vary according to the organs or tissues in which the cells are placed: besides, it does not seem improbable that, in the case of the cells of the intestinal villi, their own growth may be effected by means of the materials which they absorb from the chyme for the formation of chyle, and thus may be independent of the blood-vessels; for it does not appear that they ever grow, except when chylification is going on.

The epithelial coat of the villi seems to be chiefly destined for the protection of their surface at those times when absorption is not going on, since it is in all cases cast off when the process of absorption commences; probably, also, in common with the epithelium lining the follicles of Lieberkühn, it serves some further purpose in preparing the chyme for absorption. Proof that the epithelial coat is thrown off during the passage of chyme along the intestines, removes the difficulty which it was conceived the cells would experience in effecting their absorption through the layer intervening between them and the chyme. The thin germinal membrane would be no impediment to the process of absorption; on the contrary, it would rather be favourable to it. Mr. Fenwick, of North Shields, has performed numerous experiments, and worked out very elaborately the subject of lacteal and lymphatic absorption, though the results he has attained do not help much in clearing the mystery which still to a certain extent hangs over this department of physiology.

7. *Structure of the liver.* In alluding to Müller's recent paper on the structure of the liver, in which this physiologist maintains the lobular arrangement of the component parts of this organ, Mr. Paget says, "The only point in which I think Müller is wrong, is in describing the partitions as formed of fibro-cellular tissue. If one be cut from the interior of the liver, it will be found covered on both sides with hepatic cells and granules, which adhere to it much more firmly than those in the interior of the lobule do to one another. When these are scraped off, there remains a very thin and tough membrane, in which there are only a few filaments of fibro-cellular tissue, and which appears to be composed of a very dense network or networks of vessels, with gland cells still adhering among them. The appearances presented in the pig's liver are such as to indicate that its lobules are by no means generally or uniformly traversed by plexuses of ducts; in their interior they appear to contain only large nucleated biliary cells, with various granules loosely arranged; the ducts appear only in the walls of the lobules."

8. *Functions of the Bile.* Following is Mr. Paget's analysis of the recent experiments by Schwann, relating to the functions of the bile. "The experiments lead to the distinct conclusion of the bile being indispensable to life. They consisted in removing a portion of the common bile duct, and establishing an external fistulous opening into the gall-bladder, so that the bile might be naturally secreted, but be discharged externally, and not permitted to enter the intestine. Their general result was, that of eighteen dogs thus operated on, ten died of the immediate consequences of the operation, and of the remaining eight, two recovered and six died. In the six which died, death was the result of nothing but the removal of the bile; after the third day, they daily lost weight, and had all the signs of inanition; e. g., emaciation, muscular debility, uncertain gait, falling of the hair. They lived from seven to sixty-four days after the operation; and the inanition was the greater the longer they survived. Young dogs appeared to die rather sooner than old ones. Licking the bile as it flowed from the fistula, and swallowing it had no influence on the consequences of the operation. In the two dogs that recovered, the importance of the bile was equally well shown; for, on these being examined, it was found that the passage for the bile into the intestine had been restored; and the period of its restoration was distinctly marked by their weight (which had previously been regularly decreasing) being augmented and continuing to increase till it amounted to what it was before the operation; and also by the fistulous opening into the gall-bladder healing, and the discharge of bile ceasing.

### § III.—*Circulation.*

9. *Venous Pulse.* M. Martin Solon has given the details of two cases in which he observed pulsation of the dorsal veins of the hands. The patients had both been repeatedly bled, and taken tartar emetic, for an attack of pleuro-pneumonia. The veins were prominent, rounded, of a blueish-red colour, and presented a diastolic and systolic movement, easily appreciated by the eye, and synchronous with the pulse: it was evidently not communicated by any adjacent vessels. Upon pressing the fingers, the pulsations ceased; but when the wrists were pressed they remained as before. When the brachial artery was pressed, the pulsation of the radial and ulnar arteries, and of the dorsal veins of the hand, all disappeared together. In both cases, the patients gradually recovered. In one the venous pulsation appeared on the fifteenth day, and remained seven days, the cardiac impulse being strong: in the other, the heart's impulse was feeble, and the venous pulsation remained for a shorter time. M. Solon explained the phenomenon of venous pulsation in these cases, by supposing that the abnormal fluidity of the blood facilitated its passage through the capillaries, and thus enabled it to retain the impulse communicated by the heart. He alluded to similar cases by Dr. Graves and Dr. Ward. Pathologically, he thought the phenomenon important, as indicating a state of fluidity of the blood, which would render further bleeding inadvisable. Physiologically, it was important, as proving that the entire circulation is under the influence of the heart. In a discussion which ensued after the reading of the memoir, M. Poiseuille agreed with Mr. Solon in considering the phenomenon as another proof of the influence of the heart over venous circulation, but could not attribute it to the greater fluidity of the blood, for the experiments of Magendie and himself had proved that the more aqueous the blood became, the greater was the difficulty with which it passed through the capillaries, owing to imbibition. He thought it, therefore, more correct to explain the influence which loss of blood evidently had in producing venous pulsation, by considering the heart as having lost energy, whereby a smaller quantity of blood is thrown into the arteries, which being less dilated, contract with less force, and thus lose their power of converting the intermittent fluid into a continuous one, as is normally the case.

10. *Moving powers of the blood.* Dr. Calvert Holland, in a recent work on the circulation, has raised objections to most of the now generally admitted truths respecting the agencies concerned in carrying on the circulation, as established by the experiments of Sir David Barry, Poiseuille, Magendie, and many others. Dr. Holland has displayed considerable ingenuity in enforcing his arguments, and in some particulars he is correct; but, on the whole, his objections are not sufficiently weighty to entitle their being received as valid. He has dealt so much at length with his arguments, that even an abstract of them cannot be given here.

11. *Agency of the pericardium in the circulation.* Mr. J. H. Walshe, of Worcester, has offered an ingenious theory to explain how that imaginary difficulty, the return of venous blood to the heart, contrary to gravity, may be overcome. He considers the pericardium, by virtue of its structure and arrangement, to be the chief agent, (through the medium of the ventricles,) by which this object is effected. He compares the human pericardium to an unyielding tent or box, and considers it so firmly fixed at its several points of attachment, as to be capable of resisting considerable pressure from without, and of not yielding to the various movements of the inclosed heart. Assuming this arrangement to be true, and the cavities of the heart with their contained blood always to occupy a given space, which is maintained of the same dimensions by the pericardium, Mr. Walshe reasonably infers that, as the ventricular contraction forces out a certain amount of blood, an equivalent proportion must rush into the auricles, otherwise a space would exist within the unyielding pericardium, resulting from the contraction and emptying of the ventricles. In this way, he revives the old notion, which attributed to the ventricles a sucking as well as a propelling power. This ingenious theory will hardly be found reconcilable with facts. The human pericardium and its attachments are more yielding than Mr. Walshe admits; witness the large effusions occasionally contained within its cavity, the great displacement of the heart and pericardium induced by pleuritic effusions, emphysematous lungs, &c. Again, the pericardium must be somewhat yielding to allow of its parietal surface following the sternum upwards and forwards, and the diaphragm downwards, during inspiration; for the great vessels at the base, to which the pericardium is attached, form a fixed unyielding point. The existence of this amount of flaccidity renders it pretty certain that the pericardium and surrounding soft textures will readily fall in, and fill up the space left by either the ventricular or auricular contraction. To these, other arguments might be offered, as the existence of an adherent pericardium without consequent hypertrophy of the heart; one or two instances of a heart without a pericardium; but the above are sufficient to render doubtful this new attribute of the pericardium, the advantage of which, indeed, were it to exist, would be trifling, for the amount of force expended by the ventricles in suction would be of greater ultimate advantage if entirely directed to the onward current. The difficulties attending the return of venous blood to the heart are, moreover, greatly overrated.

Mr. Walshe assigns another useful purpose resulting from this assumed arrangement of the pericardium, that of entirely preventing regurgitation from the auricles, and thus dispensing with valves at the mouths of the large veins. This again is hardly tenable, for auricular regurgitation does to a certain extent take place, as shown in the experiments of Barry and Poiseuille, where, during the diastole, fluid rose in the tube inserted into the jugular vein: further regurgitation is prevented by the contraction of the muscular fibres which surround the mouths of the large veins opening into the auricles.

### § IV.—*Respiration.*

12. *Physiology of asphyxia.* Mr. Erichsen, in a recent admirable essay, states that the cause of the stoppage of the circulation in asphyxia is threefold, depending:—1st. Upon the arrest of the respiratory movements; the influence of this agent is comparatively trifling.—2d. Upon the weakening of the heart's action; this has an important influence in arresting the circulation. The enfeebled condition of the heart results from the circulation through its muscular substance of blood deprived of its stimulating arterial qualities, and diminished in quantity. When a due supply of arterial blood is furnished to its muscular substance, the heart is enabled to propel even black blood through a lung entirely deprived of air; this, Mr. Erichsen proved by an experiment of tying the right bronchus of a dog, at the same time keeping up artificial respiration through the left lung ; in nine minutes after the ligature of the bronchus, he tied one of the pulmonary veins of the same side, and then punctured it on the distal side of the ligature. Black blood flowed from the puncture, and continued to ooze for eight minutes, when the experiment was discontinued. During the whole time not a bubble of air gained admission into the right lung, (consequently, all chemical changes in it must have ceased,) yet black blood continued to circulate through it; during this time the heart was supplied with arterial blood from the left lung.—3d. Upon obstruction offered to the passage of venous blood through the lungs. This results not from paralysis of the minute pulmonary vessels, in consequence of their proper "nervous influence" being destroyed by the circulation of venous blood through the medulla oblongata, for Mr. Erichsen proved that the circulation ceases as quickly in an animal whose trachea is closed, when arterial blood from another animal is supplied to its brain and medulla oblongata, as when these parts are permeated by venous blood. Neither, again, does this obstruction depend upon the mere arrest of chemical changes between the air and blood, for these changes cease in two minutes after closure of the trachea, whereas the circulation continues, and the femoral artery pulsates for six minutes longer. The cause of this obstruction in the pulmonary circulation, advocated by Mr. Erichsen, is the refusal of the minute pulmonary veins (not the capillaries) to receive venous blood, which stimulates them to contract upon their contents. This action is contrary to what the general sedative properties of venous blood would lead us to expect; but he explains it by reference to the examples of substances acting as *sedatives* to one surface and *stimulants* to another, although the surfaces may not present any appreciable difference in structure. In a similar manner, the minute systemic arteries contract upon and obstruct the flow of venous blood through them, thus offering a further obstacle to the already enfeebled heart.

These various impediments to the heart's action increasing, especially the circulation of venous blood through its muscular substance, its contractions cease, and with this cessation occurs the extinction of organic life, which usually takes place under ten minutes from the commencement of the asphyxiating cause; although perfect insensibility and loss of voluntary movement occur in about one and a half or two minutes, in consequence of the circulation of unoxygenized, unstimulating blood through the brain; the medulla oblongata preserves its functions somewhat longer.

13. *Minute structure of the lungs.* The following abstract of a paper lately read by Mr. Rainey, before the Medico-Chirurgical Society, is taken from the *Medico-Chirurgical Review.*

1st. A bronchus, when traced from its commencement to its termination, is seen to be, in the first part of its course, more or less cartilaginous; it then becomes destitute of cartilage, retaining, however, a perfectly circular form, and having no air-cells opening into it; farther on, being still circular, numerous air-cells open into it; lastly, the air-cells increase so much in number, and open into the bronchus so closely to one another, that the tube can no longer retain its circular form, but becomes reduced to an irregular passage, running between the cells, and ultimately reaching the surface of the lobule, ends by forming a terminal air cell.

2d. The air-cells are small irregularly-shaped cavities, having generally four or five unequal sides; those which are situated close to the small bronchial passages open into them by well-defined circular apertures, whilst those which are situated at a distance from these passages open one into the other—an arrangement which those who are acquainted with the disposition of the air-cells in the injected lung of the frog and serpent will readily comprehend; in fact, each lobule of the lung of the mammal and man, with its bronchial passages and appended cells, may in some sort be regarded as a repetition of the whole lung of the frog.

3d. The border of each air-cell is surrounded, in addition to the epithelium, by a number of fibres definitely arranged in a circular manner, so as to form a circumscribed limit to each cell. The fibres appear to be elastic, and have no resemblance whatever to muscular fibres, striped or unstriped.

4th. The sides or walls of the air-cells consist of a dense plexus of capillaries, situated in the interior of the lobules, between two layers of the pulmonary membrane; but, on their exterior, between this membrane and the pleura, in the case of the lobules on the outer surface of the lung, or between it and the interlobular areolar tissue in those lobules which bound the interlobular spaces. There is thus, between every two cells, only one vascular network, so that the small stream of blood in each capillary vessel is acted on by the air upon both sides; whilst, in the frog, serpent, &c., there being two plexuses of vessels between two cells, the blood in the capillaries is only aerated on one side.

5th. The number of capillary plexuses is not the same as that of the air-cells, one network passing between and supplying several cells; or, in other words, one terminal branch of the pulmonary artery supplies the plexuses of several air-cells.

6th. The fœtal lungs, prior to the act of respiration, when well injected, are distinctly seen to possess air-cells, fully formed and surrounded, as in the animal which has respired, by plexuses of blood-vessels.

M. Rochoux's account, so far as it goes, corresponds with the above accurate description given by Mr. Rainey. According to M. Rochoux's calculation, the number of pulmonary cells amounts to about 600,000,000, and there are about 17,790 grouped around each terminal bronchus.

### § V.—*Structure and Formation of Tissues.*

14. *Structure of lymphatic glands.* Some information concerning the internal structure of lymphatic glands has been afforded by Mr. Goodsir. The interior of each lymphatic and lacteal gland is made up of anastomosing ramifications of the afferent and efferent lymphatic vessels—these anastomosing branches are arranged very closely together, their external surfaces being separated from each other by a very fine capillary network formed by the blood-vessels of the gland. The extra glandular lymphatics as they enter the gland lose their external filamentous coat, which becomes continuous with the capsule of the gland—their middle fibrous coat also disappears shortly after the vessels have entered the gland. The internal coat is therefore the only remaining texture of the intra-glandular lymphatics; this coat becomes greatly thickened and opaque. It consists of a fine transparent external membrane, containing ovoidal nucleated cells (germinal spots) placed at regular distances along it. Within this external membrane and almost filling up the canal of the lymphatic vessel, is a thick layer of nucleated epithelial particles; this layer is thickest in the lymphatics towards the centre of the gland, and gradually diminishes towards the circumference, where it becomes continuous with the layer of flat epithelium scales of the extra-glandular lymphatics. The epithelial particles are on an average about $\frac{1}{5000}$ of an inch in diameter. They are probably being constantly (though with periodically increased activity as during the passage of lymph or chyle) developed from the germinal spots of the external membrane, and when formed they probably discharge some important function towards the lymph or chyle contained in the canal, and with which they are always in contact.

15. *Secreting structures.* The following comprises an abstract of the chief points contained in an excellent paper by Mr. Goodsir; the subject relates to the function of secretion as well as to the structure of secreting organs, and is in many respects original.

1st. Secretion is essentially a function of nucleated cells. The cells endowed with this property of secretion possess a peculiar organic power by which they can draw into their interior certain kinds of materials varying according to the nature of the fluid they are destined to secrete. Some cells have merely to separate certain ingredients from the surrounding medium, others have to elaborate within themselves matters which do not exist as such in the nutritive medium.

2d. Though secreting cells thus differ in the nature of the fluid which they secrete, (as whether milk, bile, saliva, or other,) their structure seems to be nearly the same in all cases; each consisting, like other primitive cells, of a nucleus, cell-wall, and cavity.

3d. The nucleus seems to be both the reproductive organ by which new cells are generated, and the agent for separating and preparing the secreted material. The cell-cavity seems destined chiefly to contain the secreted fluid until ready to be discharged, at which time, the cell then matured, bursts and discharges its contents into the intercellular space in which it is situated, or upon a free surface, as the case may be.

4th. The mode of secretion in glands of which the testicle of the *squalus cornubicus* may be taken as a type, seems to be the following. Around the extremities of the minute ducts of the glands are developed acini or primary nucleated cells, each of which as it increases in size has generated within it secondary cells, the product of its nucleus. The cavity of the parent cell does not communicate with the duct on which it is situated until its contents are fully matured, at which time the cell-wall bursts or dissolves away, and its contents are discharged into the duct. From this constant succession of growth and solution of cells, it results that the whole parenchyma of a gland is continually passing through stages of development, maturity, and atrophy, the rapidity of which process is in proportion to the activity of the secretion. There seems, therefore, to be no essential difference between the process of secretion and the growth of a gland, the same cells are the agents by which both purposes are effected; the parenchyma of glands is chiefly made up of a mass of cells in all stages of development; as these cells individually increase in size and so constitute their own growth as well as that of the common glandular mass, they are at the same time elaborating within themselves the material of secretion, which, when matured, they discharge, by themselves dissolving away. There are a number of germinal spots or centres in a gland from which acini or primary cells are developed.

5th. The true fluid of secretion is not the product of the parent-cell of the acinus, but of its included mass of secondary cells, which themselves become primary secreting cells, and form the material of secretion in their cavities. In some cases these secondary cells pass out entire from the parent-cell, constituting a form of secretion in which the cells possess the power of becoming more fully developed after being discharged and cast into the duct, or cavity of the gland.

6th. In the order of glands, which consist of follicles more or less elongated, the following is the arrangement:—At the blind extremity of each follicle is situated a germinal spot, at the centre of which is constantly or periodically developing nucleated cells. These cells, as they become developed, tend towards the open extremity of the follicle. At first they are simple nucleated cells, but as they advance they gradually assume the characters of primary secretory cells, and contain secondary cells in their interior. When fully matured and arrived at the attached extremity of the follicle, the primary cells burst and allow their contents to pass into the branch of the duct to which the follicle is attached. Each follicle is virtually permanent, though both its contained cells and its walls are continually undergoing change, receiving development and addition at the blind extremity, being absorbed and disappearing at the other.

7th. Mr. Goodsir considers that the process of original development of glands in the embryo is identical in its nature with the growth of a gland during its state of functional activity. The blastema which announces the approaching formation of a gland in the embryo, in some instances precede, and is in other instances contemporaneous with, the conical blind protusion of the membrane upon the surface of which the future gland is to pour its secretion. In certain instances it has been observed that the smaller branches of the ducts are not formed by continued protrusion of the original blind sac, but are hollowed out, independently, in the substance of the blastema, and subsequently communicate with the ducts. It appears highly probable, therefore, that a gland is originally a mass of nucleated cells, the progeny of one or more parent-cells, and that whether the membrane in connexion with the embryo of the gland sends a conical protrusion into the mass or not, the extremities of the ducts are formed as closed vesicles, and then nucleated cells are formed within them, and are the parents of the epithelium cells of the perfect organ.

16. *Structure of serous membranes.* Mr. John Goodsir offers the following observations on the structure of serous membranes. A portion of the human pleura, or peritoneum, consists, from its free surface inwards, of a single layer of nucleated scales ; of a germinal membrane, (vide account of, by Mr. Goodsir ;) and of the sub-serous areolar texture intermixed with occasional elastic fibres. The blood-vessels of the serous membrane ramify in the areolar texture. The germinal membrane seldom shows the lines of junction of its component flattened cells. These appear elongated in the form of ribands, their nuclei, or the germinal spots of the membrane, being elongated, expanded at one extremity, pointed at the other, and somewhat bent upon themselves ; they are bright and crystalline, and may or may not contain smaller cells in the interior. If these germinal centres be the sources of all the scales of the superficial layer, each centre being the source of the scales of its own compartment, then the matter necessary for the formation of these during their development must pass from the capillary vessels to each of the centres, acted on by forces whose centres of action are the germinal spots ; each of the scales, after being detached from its parent centre, deriving its nourishment by its own inherent powers.

17. *Structure of bone.* Mr. Goodsir describes the soft portion of bone to be situated within the bone-corpuscles, and to consist in each corpuscle of a little mass of nucleated cells of great transparency ; the soft portion, therefore, is not continuous like the hard, but is divided into as many portions as there are corpuscles.

2. The hard portion of bone is made up of cells filled with bony substances, and ossified or calcified primordial cells, which cells as they grow old are constantly dissolving, and their debris falling back into the returning circulation, whilst new ones are being formed from the mass of nucleated cells (which are so many centres of nutrition;) within the bone-corpuscles. Each of these centres of nutrition is constantly absorbing nutritive materials from the blood-vessels through the medium of the calcigerous canals, and appropriating them partly for the nourishment of the existing calcigerous cells, but more especially for the formation of new ones, which it supplies to all parts within its own territory, or within the range of its own system of calcigerous canals.

3d. The hard and soft parts of bone, which when combined constitute the true osseous texture, only differ from each other in this, that the cells of the one are ossified ; those of the other retain their original delicacy and softness.

4th. In every true bone their exists, between the blood-vessels and the walls of the Haversian canals, a layer of cellular substance, the cells of which Mr. Goodsir considers to be the descendants of the corpuscles of the cartilage or matrix in which the bone was originally formed; for in the original development of the bone around each cartilage-corpuscle, are formed a number of secondary corpuscles which arrange themselves in a linear series, the rows of which assume a direction perpendicular to the ossifying surface. These secondary cells remain as centres of nutrition for the future bone, whilst the progeny formed from each constitutes a cellular mass; around each such cellular mass, the capsules, or laminæ, of compact primary bone become formed, and when these capsules have opened into each other, they form Haversian canals, the blood-vessels within which are separated from the walls of the canals by the layer of cellular substance. The great extent of, and the prominent position held by this cellular substance in the development of bone, proves that it must play some important part in bone economy. In adult bones this cellular substance is, in the medullary cavity, cancelli, and to a certain extent, in the larger Haversian canals, replaced by fat-cells.

*Relative properties of animal and earthy matters in bone.* Dr. Stark has made some very numerous analyses of bones, chiefly with the view of determining the proportional amounts of earthy and animal matters in the bones of the different classes of vertebrate animals. He has examined human bones, and those of an extensive number of mammalia, birds, reptiles, and fishes, and has given a comprehensive table showing the results. The most interesting conclusions to be derived from these experiments, are:—1st. The proportion of earthy and animal matters in the bones varies but little over the whole animal kingdom; wherever a true bone occurs, that bone contains nearly the same average amount of earthy and animal matters; therefore, the statement that the higher we ascend in the scale of organization, the larger amount of earthy matters do the bones contain, is fallacious. 2d. The animal matter composes about one-third of the weight of the dry clean bone: thus the mean proportion in the bones of all vertebrate animals 66·09 per cent. of earthy, 33·91 of animal matter; the mean proportion in the bones of man is 66·61 earthy, 33·39 animal matter. The proportion of earthy matters in the bones of wild mammalia seems to be a fraction higher than in domesticated animals. 3d. Age does not seem to increase the amount of earthy matters in the bones, as is generally supposed. 4th. The hardness of bone does not depend on the amount of earthy matter contained in it, as is shown in the readiness with which the bones of fish may be cut, although they contain as large an amount of earthy matter as the ivory-like leg bones of the deer or sheep. 5th. Neither increased flexibility, nor transparency of bones (as in the bones of fish) depends on a diminished proportion of earthy solids in its texture; but like that of increased hardness, probably on the peculiar structural arrangement of the tissue. The great fault in the analysis of bones hitherto published is, that the amount of animal matter has been rated too high, probably from want of care in drying the bones and properly freeing them from fat or oil previous to burning. Dr. Stark alludes to the excessive fragility of human bones as contrasted with those of animals, so that when prepared for chemical examination and deprived of their membranes and fat, they may be readily crushed by firm pressure between the finger and thumb; whereas, the bones of the lower animals similarly prepared, will bear the roughest handling without injury. He suggests that this circumstance might account for the fact of human bones never being met with in those tertiary deposits in which the bones of lower animals are so abundant.

18. *Vessels in fat, smaller than capillaries.* Mr. Smee describes a new set of minute vessels existing as appendages to fully developed fat, and to which he gives the name of "Vasa adipis." These minute vessels measure from about $\frac{1}{10000}$ to $\frac{1}{25000}$ of an inch in diameter; they are given off from the capillaries and are distributed at every angle of each fat-cell. They exist only in fat, the globules of which have assumed their polygonal form. Although the term vessel is applied to these structures, yet Mr. Smee states that no evidence whatever can be adduced either of the existence in them of a cavity or of distinct walls, beyond their being permeable by fluid injections, [which in ordinary cases would indicate the existence of a cavity.] Mr. Smee suggests nothing as to their function, but states that they must not be considered as vasa serosa which system of vessels are, in all probability, quite imaginary. [The existence of these vasa adipis requires further confirmation.]

19. *Centres of nutrition.* Mr. Goodsir has recently made several important additions to the doctrine of cell-formation. Amongst other observations he states that, besides all organs and tissues having their origin in, and consisting essentially of simple or developed cells possessed of a peculiar independent vitality, these component cells are moreover divided into numerous departments, each of which consists of several cells arranged round one central or capital cell, which latter is the source whence all the other cells, in its own department, have derived their origin. To each of these several central nucleated cells, he applies the name of *nutritive centre*, or germinal spot. Each nutritive centre possesses the power of absorbing materials of nourishment from the surrounding vessels, and of generating, by means of its nucleus, successive broods of young cells, which from time to time fill the cavity of the parent cell, and carrying with them its cell-wall, pass off in certain directions and under various forms, according to the texture or organ of which their parent forms a part. There are two kinds of nutritive centres, those which are peculiar to the textures, and those which belong to the organs. The former are in general permanent, the latter are mostly peculiar to the embryonic state, and ultimately disappear. There is one form in which the nutritive centres are arranged, both in healthy and morbid parts, which constitutes what Mr. Goodsir calls a *germinal membrane;* it is only met with on the free surface of organs or parts: it is a fine transparent membrane, and consists of cells arranged at equal and variable distances within it; the cavities of these component cells are flattened, so that their walls form the membrane by cohering at their edges, and their nuclei remain in its substances as the germinal centres. One surface of the membrane is attached to the surface of the organ of part, and is therefore applied upon a more or less richly vascular tissue; the other surface is free, and it is on it only that the developed or secondary cells of its germinal spot are attached. These secondary cells, whilst forming, are contained between the two layers of the germinal membrane, but, as they become fully developed, they carry forward the anterior layer and become attached to the free surface, whilst the nuclei are left in the substance of the posterior layer, in close contact with the blood-vessels from which they derive the materials for the formation of new cells.—*Ranking's Half Yearly Abstract.*

[*To be continued.*]

